# First-Principles Study on Strain-Induced Modulation of Electronic Properties in Indium Phosphide

**DOI:** 10.3390/nano14211756

**Published:** 2024-10-31

**Authors:** Libin Yan, Zhongcun Chen, Yurong Bai, Wenbo Liu, Huan He, Chaohui He

**Affiliations:** 1 Department of Nuclear Science and Technology, Xi’an Jiaotong University, Xi’an 710049, China; yanlb22@stu.xjtu.edu.cn (L.Y.); chenzhongcun@stu.xjtu.edu.cn (Z.C.); baiyur@stu.xjtu.edu.cn (Y.B.); hechaohui@xjtu.edu.cn (C.H.); 2 China Nuclear Power Technology Research Institute Co., Ltd., Shenzhen 518000, China

**Keywords:** indium phosphide, density functional theory, strain-induced modulation, bandgap, electron effective mass

## Abstract

Indium phosphide (InP) is widely utilized in the fields of electronics and photovoltaics due to its high electron mobility and high photoelectric conversion efficiency. Strain engineering has been extensively employed in semiconductor devices to adjust physical properties and enhance material performance. In the present work, the band structure and electronic effective mass of InP under different strains are investigated by ab initio calculations. The results show that InP consistently exhibits a direct bandgap under different strains. Both uniaxial strain and biaxial tensile strain exhibit linear effects on the change in bandgap values. However, the bandgap of InP is significantly influenced by uniaxial compressive strain and biaxial tensile strain, respectively. The study of the InP bandgap under different hydrostatic pressures reveals that InP becomes metallic when the pressure is less than −7 GPa. Furthermore, strain also leads to changes in effective mass and the anisotropy of electron mobility. The studies of electronic properties under different strain types are of great significance for broadening the application of InP devices.

## 1. Introduction

As a III–V semiconductor material, indium phosphide (InP) exhibits outstanding optoelectronic properties, including a direct bandgap, high electron mobility (4600 cm^2^∙V^−1^∙s^−1^) and excellent photoelectric conversion efficiency (22.1%) [1]. Therefore, InP-based∙ materials are widely utilized in high-speed optical fiber links, optical amplifiers, modulators and solar cells [2,3].

Strain engineering is a powerful physical property modulation technique that can lead to changes in various properties of crystals, such as bandgap transition [4,5,6], phase transition [7], light absorption intensity [5,8,9], etc. It is widely used to improve the performance of electronic and photonic devices [10,11,12,13]. For example, strained silicon technology has been extensively adopted in Complementary Metal–Oxide–Semiconductor (CMOS) devices to improve carrier mobility and, consequently, the conductivity through the channel region [14]. Therefore, in order to better understand the properties of materials, it is important to study changes in the electronic properties of semiconductor compounds under different strains.

Currently, strain engineering has been widely applied in InP and InP-based materials. Kim et al. investigated the electronic band structure and effective mass of InP and its alloys under biaxial strain [15]. Mondal et al. examined the effect of uniaxial, biaxial and isotropic strains on the bandgap of InP [16,17]. Kabita et al. explored the structural, elastic and electronic properties of InP in sphalerite and rock salt phases under different pressures, discovering that a pressure-induced structural phase transition from sphalerite to rock salt occurred at 9.3 Gpa [18]. Branicio et al. studied the high-pressure phase of InP and found that the phase transition from Zinc Blende (ZB) to rock salt (RS) occurred at a pressure of 10.2 Gpa [19]. These studies collectively demonstrate the promising advantages of strain engineering in enhancing the performance of InP-based devices, but they do not provide a comprehensive analysis of the trend of effective electronic properties of InP under different strains. It is crucial to study the effect of strain on the effective mass of electrons, as this property can affect the electronic conductivity of materials and ultimately affect device performance.

In this paper, we discussed the study of the band structure and the electronic effective mass of indium phosphide (InP) under different strains, using ab initio calculations to analyze the effects of strain on bandgap values. We also explored the metallic properties of InP under different hydrostatic pressures and the impact of strain on effective mass and the anisotropy of electron mobility. Our findings provide useful information on fully unlocking the potential of strain-engineered InP for power electronics and optical applications.

## 2. Materials and Methods

**Figure 1:**
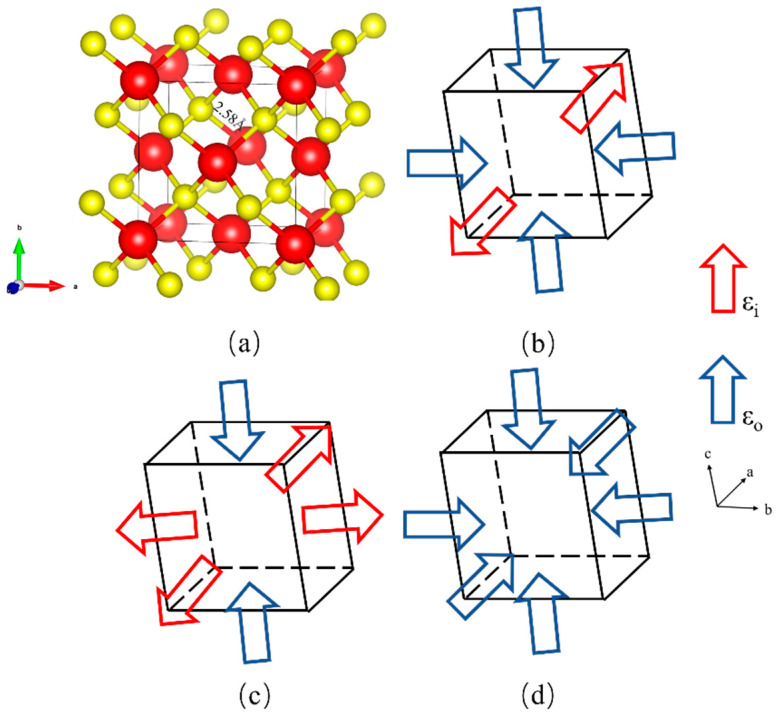
(**a**) The conventional unit cell of InP. Schematic illustrations of (**b**) uniaxial, (**c**) biaxial and (**d**) hydrostatic pressure. The red balls denote In atoms, and the yellow balls denote P atoms. The red and blue arrows represent induced strain ($\varepsilon_{i}$) and optimized strain ($\varepsilon_{0}$), respectively.

All simulations were performed by density functional theory (DFT) [20] implemented in the QuantumATK software package of version 202312-SP1 [21]. The exchange–correlation energies were processed within the generalized gradient approximation (GGA) in the form of the Perdew–Burke–Ernzerhof (PBE) [22]. We utilized the PSEUDODOJO pseudopotential with a high basis set to accurately extend the electron density [23], and a planewave cut-off energy of 78 hartree was used for all structures. The energy difference was converged to 10^−5^ eV and the maximum residual force was converged to 0.01 eV/Å. Prior to the electronic structure calculations, geometry optimization was conducted. The number of Monkhorst–Pack k-point sampling points [24] in the Brillouin zone was 8 × 8 × 8 for the structural relaxation of the conventional cell containing 8 atoms. Figure 1a shows the conventional unit cell of InP. The calculated InP exhibits a sphalerite structure. The lattice parameter is 5.968 Å, which is in good agreement with previously reported values of 5.869 Å [25].

Three types of strains, uniaxial strain, biaxial strain and hydrostatic pressures, are applied in InP material, as shown in Figure 1b–d. The strain applied in the a-direction is defined as $\varepsilon_{a}=(a-a_{0})/a_{0}$, where $a_{0}$ represents lattice constants in the strain-free state. Uniaxial strain is simulated by applying strain along the a-axis while relaxing the other two directions. Biaxial strain is simulated by straining the two directions, a and b, and then relaxing the c-direction. The strain ranges from −10% to 10% in steps of 1%, resulting in a total of 21 data points.

For bandgap calculations, the accurate Heyd–Scuseria–Ernzerhof (HSE06) hybrid functional was adopted. To obtain an accurate bandgap, the method of k-path was chosen, and a total number of 200 points was used here. The choice of the number of k-points can be seen in Appendix A. The screening parameter of the α value [26] in the HSE simulation was set at 0.44 to obtain a bandgap value of 1.427 eV, which is in good agreement with the experimental value of 1.42 eV [27].

Regarding the calculations of strain dependence, we evaluated the strain dependence of effective masses in the primary conduction band (CB) valleys. The carrier effective mass ($m^{*}$) is shown in Equation (1).

(1) $$\frac{1}{m^{*}}=\frac{1}{{ћ}^{2}}\frac{\partial^{2}E}{\partial k^{2}}$$

where $m^{*}$ is the effective mass, *ħ* is the reduced Planck constant, *E* is the energy and *k* is the wave vector in the reciprocal lattice. The values of $m^{*}$ were obtained by fitting the energy dispersion of the conduction band minimum to the parabola function in different *k*-directions near the Γ point.

## 3. Results

### 3.1. Uniaxial and Biaxial Strain

#### 3.1.1. Lattice Structure

**Figure 2:**
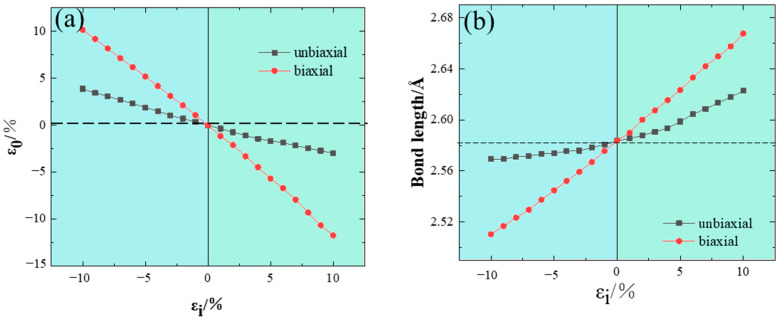
(**a**) Optimized strain values and (**b**) bond lengths of In–P under different strain states. Uniaxial and biaxial correspond to the induced strain values. The dashed lines indicate the parameters of In–P without any strain.

Before the calculations of electronic properties, the structure relaxation of strained InP was performed. The results of optimized strain are shown in Figure 2a. It is observed that the optimal strain decreases with the increment in both the corresponding induced uniaxial and biaxial strain models.

Notably, the change in the optimized strain under uniaxial strain is significantly less than that observed under biaxial strain. Furthermore, the variation trend for the optimized strain is opposite to that for the induced strain, which is consistent with the Poisson effect. The Poisson’s ratio calculated from our uniaxial strain model is 0.35, which aligns well with the previous experimental value of 0.36 [28]. Since the Poisson ratio of biaxially strained InP is higher, it is reasonable that the change in structural parameters under biaxial strain is more pronounced.

In addition, the changes in bond length caused by these two types of strain were also calculated, as depicted in Figure 2b. Within the studied strain range, the change in bond length between In–P is roughly proportional to the induced strain. The biaxial strain has a more pronounced effect on the bond length. At 10% uniaxial strain, the key length is 2.62 Å, while for biaxial strain, the key length is 2.67 Å at 10% strain.

#### 3.1.2. Band Structure

**Figure 3:**
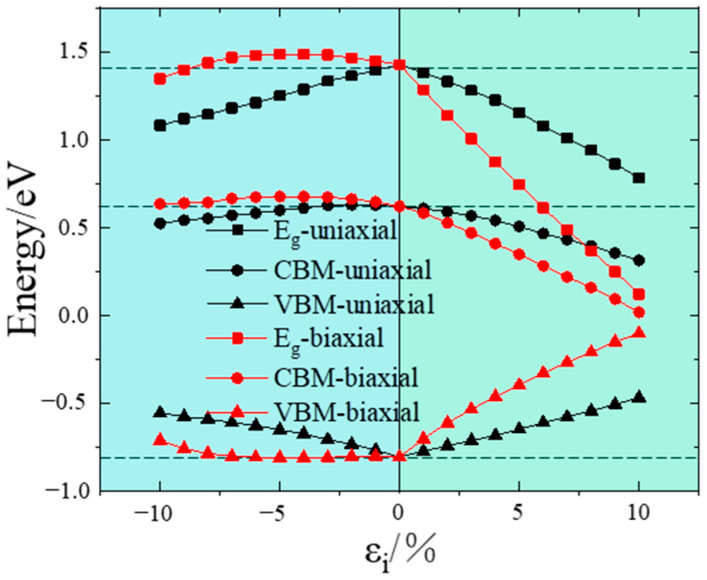
Strain-induced effects on the bandgap value of InP as functions of uniaxial and biaxial strain. $E_{g}$ represents the difference between the VBM and CBM; the VBM and CBM represent the valance band maximum and conduction band minimum, respectively.

To gain a comprehensive understanding of the electronic band structure behavior under strain, the strain-induced changes in bandgap values were further calculated, as displayed in Figure 3. This figure illustrates the strain dependence of the valence band maximum (VBM), the conduction band minimum (CBM) and the energy difference, $E_{g}$ ($E_{g}$ = CBM − VBM), for the uniaxial and biaxial strain.

Under biaxial compressive strain, the bandgap initially increases from 1.427 eV to 1.486 eV and then monotonically decreases from 1.486 eV to 1.346 eV. The maximum bandgap occurs at approximately $\varepsilon_{i}$ = −5%. Conversely, under biaxial tensile strain, the bandgap decreases monotonically, continuously dropping from 1.427 eV to 0.120 eV. For the same magnitude of biaxial compressive and tensile strains, the changes in the bandgap are distinct. In the case of uniaxial strain, whether compressive or tensile, the bandgap decreases monotonically as the strain increases. Moreover, when analyzing the VBM and the CBM, it is observed that the trend in bandgap change is primarily affected by the VBM under uniaxial compressive strain. In contrast, this trend is mainly driven by the CBM under biaxial compressive strain. For tensile strain, both the CBM and VBM contribute to the bandgap trend. A comparison of uniaxial and biaxial strains reveals that uniaxial compressive strains show greater variation, and biaxial tensile strains exhibit more significant variation. This indicates that uniaxial strain has a stronger influence on electronic properties under compressive conditions, while biaxial strain exerts a greater effect under tensile conditions. Within the strain range considered in this study, when the relationship between the bandgap and strain is linear, the strain provides the largest adjustment range for the bandgap, resulting in the most effective tuning. Additionally, within the strain range of −10% < *ε* < 10%, InP consistently displays characteristics of a direct bandgap semiconductor, which is directly related to the larger lattice constant of InP.

The potential mechanism behind the evolution of the InP bandgap can be attributed to changes in the In–P bond length caused by strain, which ultimately affect the distance between the conduction band and the valence band. This phenomenon involves band repulsion, a behavior observed in various semiconductor and insulator materials [15,29], where the bandgap increases under compression and decreases under tension. Notably, when uniaxial or biaxial strain is applied, the light-hole (LH) band shifts upward under tensile strain, while the heavy-hole (HH) band moves upward under compressive strain [11]. This results in a reversal of the HH and LH bands at a higher compression, leading to a decrease in the bandgap as the compression strain increases.

**Figure 4:**
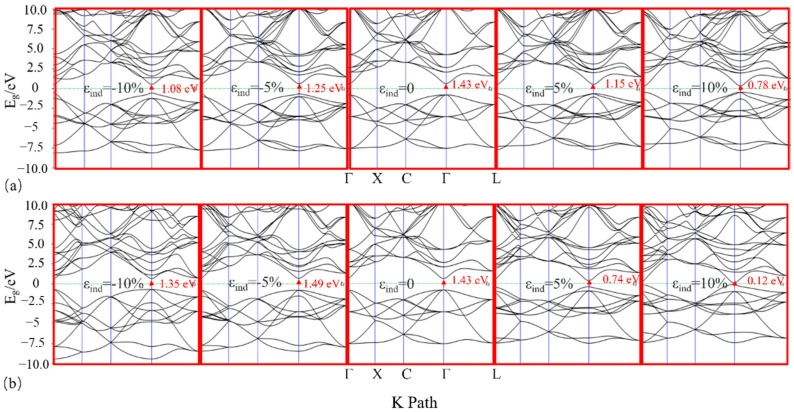
The band structure evolution of InP with respect to ±10% and ±5% (**a**) uniaxial and (**b**) biaxial strain. The bandgap value of unstrained InP (1.43 eV) is set as a reference value, the Fermi level is aligned to zero, and the arrows represent the directions from the VBM to the CBM.

To obtain a more comprehensive understanding of the changes in bandgap characteristics brought about by strain, we also examined the bandgap characteristics under various strain states. Figure 4a,b depict the band structure of InP under strains of ±10% and ±5%. The arrows indicate the direction from the VBM to the CBM. As strain increases, a detailed analysis of the band structure shows that the electron valleys at the CBM become increasingly “sharp”. Additionally, the hole valleys at the VBM also become increasingly “sharp”, although the changes are relatively smaller. This results in a narrower bandgap.

#### 3.1.3. Electron Effective Mass

Establishing a clear relationship between hole effective masses and p-type conductivity poses challenges due to the complexity and high anisotropy of hole effective masses [30,31,32,33,34,35]. To further investigate the electronic properties of InP, we only calculated the electron effective masses under various strain states. Specifically, we computed the electron effective masses along three main crystal directions (Γ-X [100], Γ-C [110], and Γ-L [111]), denoted as $m_{a}^{*}$, $m_{b}^{*}$ and $m_{c}^{*}$ respectively.

First, we compared our calculated effective mass of electrons without strain with other reported results. As shown in Table 1, the effective mass of the unstrained electrons calculated in this paper is 0.072, which demonstrates a good agreement between our findings and the reported results.

**Figure 5:**
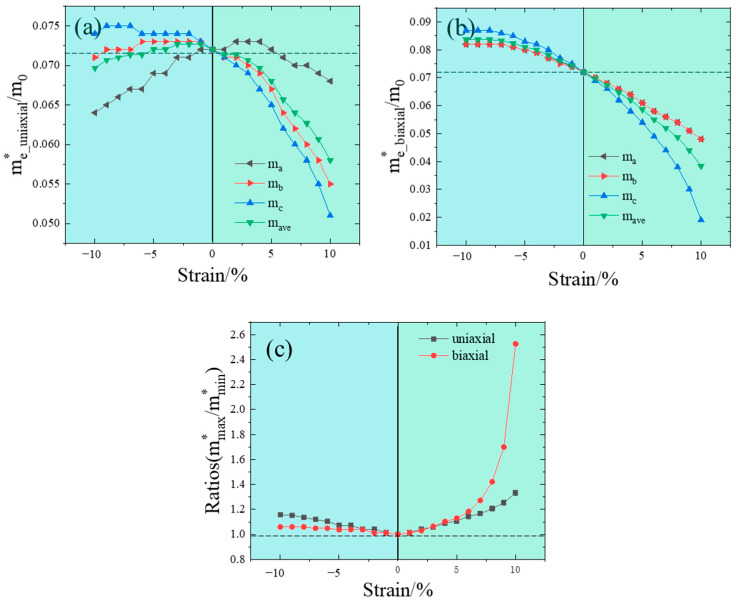
Electron effective masses of InP as functions of (**a**) uniaxial strain, (**b**) biaxial strain and (**c**) changes in the electron effective mass ratio under a different strain state. The dashed line represents the average electron effective mass and mass ratio of strain-free InP.

Furthermore, we analyzed the dependence of the electron effective mass of InP under different strain states. The corresponding results are shown in Figure 5. To demonstrate the variation in electron effective mass due to the external strain, the average value of the electron effective mass ($m_{ave}^{*}$) across three directions was taken into consideration. To qualitatively investigate anisotropy, the ratio of the maximum to the minimum values ($m_{max}^{*}/m_{min}^{*}$) was also introduced, as illustrated in Figure 5c.

A closer look at the band structure as strain increases reveals that as the bandgap narrows, the electron valley at the CBM becomes increasingly “sharper” (see Figure 4). According to k⋅p perturbation theory, the effective electron mass ($m_{a}^{*}$) at the Γ point is approximately proportional to the bandgap value. Under biaxial strain, there is almost a linear decreasing relationship between electron effective mass and strain, as shown in Figure 5b, where $m_{a}^{*}$ equals $m_{b}^{*}$, which is attributed to the isotropy of InP crystals. For uniaxial strain, a linear increase in electron effective mass is observed with compressive strain, while under tensile strain, the trend reverses. Compared with biaxial strain, the electron effective mass is slightly reduced under uniaxial strain. As illustrated in Figure 5c, the anisotropic variation under tensile strain exceeds that of compressive strain, while the isotropic change in biaxial strain surpasses that of uniaxial strain. Since the electron mobility $\mu =q\tau /m^{*}$ is determined by the effective mass of the electron, theoretically, increasing strain can enhance carrier mobility and electron anisotropy, allowing for the modulation of the electron’s effective mass and mass ratio.

### 3.2. Pressure-Induced Strain

**Figure 6:**
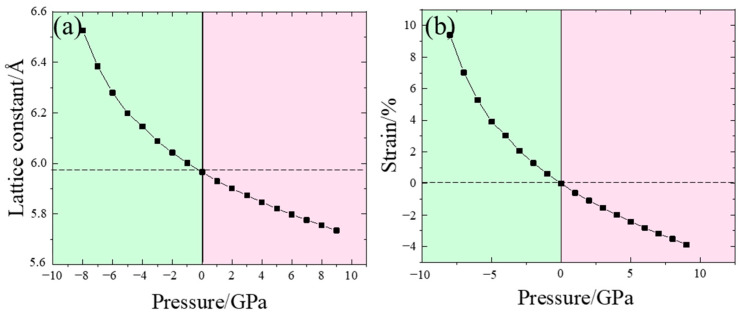
Structure parameters: (**a**) the lattice constant and (**b**) the induced strain of InP under different hydrostatic pressures.

To further investigate the effect of strain on the electronic properties of InP, we also calculated the properties of InP under hydrostatic pressure. Since InP undergoes a phase transition at pressures exceeding 9.3 GPa [18], the pressure we chose ranged from −8 to 9 GPa for our study. The structural parameters of InP at different hydrostatic pressures are shown in Figure 6a,b. From Figure 6a, it is evident that the lattice constant gradually decreases with increasing pressure. Figure 6b shows that as pressure increases, the strain progressively diminishes. The structural parameters of the crystal change significantly under applied pressure, which in turn affects the electronic structure of the material.

**Figure 7:**
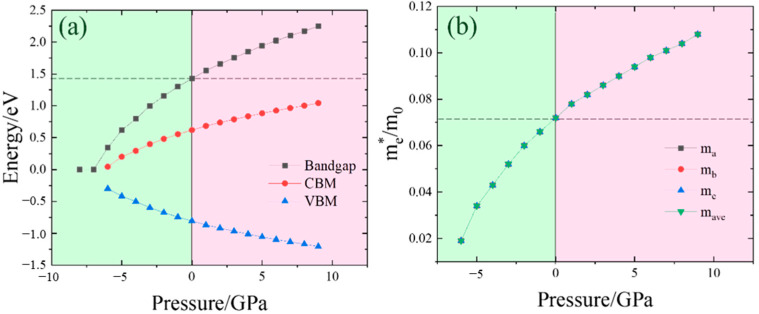
(**a**) Bandgap properties and (**b**) electron effective mass under different pressures. The VBM and the CBM represent the valance band maximum and conduction band minimum, respectively.

To more effectively study the changes in electronic structure brought about by pressure variations, we probed into the influence of pressure on the energy band. The impacts of pressure on the bandgap are displayed in Figure 7a. It is noted that as the pressure rises, the bandgap progressively increases. Simultaneously, the VBM steadily decreases and approaches stability, while the CBM gradually increases. Specifically, when the pressure increases from 0 GPa to 9 GPa, the VBM drops from −0.81 eV to −1.21 eV, and the CBM increases from 0.62 eV to 1.04 eV. Consequently, the bandgap grows from 1.43 eV to 2.25 eV. When the pressure increases from 0 GPa to −8 GPa, the VBM rises from −0.81 eV to 0 eV, and the CBM decreases from 0.62 eV to 0 eV. Thus, the bandgap decreases from 1.43 eV to 0 eV. Notably, at a pressure of −7 GPa, InP exhibits metallic properties due to the vanished bandgap. Simultaneously, the electron effective mass under different pressures was also computed. Owing to the isotropy of InP, the electron effective mass is identical in all directions. The calculated electron effective mass as a function of pressure is illustrated in Figure 7b. As the pressure gradually increases, the electron effective mass also goes up, although the rate of increase diminishes. Theoretically, increasing the pressure can enhance carrier mobility, enabling the modulation of the electron effective mass and mass ratio.

## 4. Conclusions

In the present work, we conducted a systematic study on the impact of various strains, including uniaxial, biaxial and hydrostatic pressure, on the lattice structure and electronic properties of indium phosphide (InP).

1. Under uniaxial strain, the bandgap decreases linearly as strain increases. In the calculation of the effective mass of the electron, it increases linearly with the increase in the compressive strain, but the trend is the opposite under tensile strain.
2. Under biaxial compressive strain, the bandgap initially increases and then decreases with increasing strain. For biaxial tensile strain, the bandgap decreases linearly with increasing strain. In the calculation of the effective mass of the electron, compared with biaxial strain, it is slightly reduced under uniaxial strain.
3. Under hydrostatic pressure, the bandgap increases with increasing pressure. Below −7 GPa, the bandgap value shows a metallic state. In the calculation of the effective mass of the electron, owing to the isotropy of InP, it is identical in all directions.

For all three strains, InP always maintains a direct bandgap. The calculations of effective mass indicate that the electron effective mass generally decreases with the increase in strain. Notably, the anisotropy of the electron effective mass shows an upward trend, with fluctuations under both tensile and compressive strains. Our research provides a theoretical foundation and experimental guidance for the fabrication of InP-based devices through strain engineering.

## Figures and Tables

**Table 1:** The average values of electron effective mass $m_{e}^{*}$ in InP with different functionals, where $m_{0}$ is the free electron mass.

Functionals	$m_{e}^{*}/m_{0}$
This work (HSE06) PBE	0.072 0.055 [33]
HSE06	0.089 [33]
Experiment	0.080 [36] 0.077 [37]

**Figure A1:**
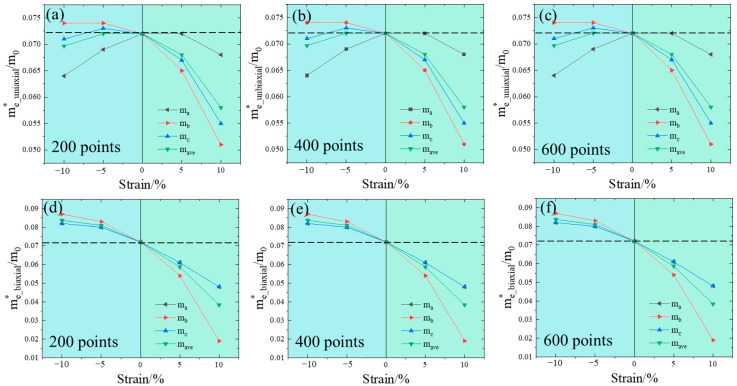
(**a**,**d**) 200 points, (**b**,**e**) 400 points, (**c**,**f**) 600 points. Electron effective mass under different numbers of points (200, 400 and 600) used in k-path sampling.

## Data Availability

The original contributions presented in this study are included in the article material, and further inquiries can be directed to the corresponding author.

## Appendix A

To verify the influence of k-point sampling, we increased the number of points used in the k-path to 400 and 600, as shown in Figure A1. Compared with the calculated results from 200 points, the electron effective mass under uniaxial and biaxial strain did not change a lot with the denser k-points, which proves that the k-point samples used in our article are accurate enough for the calculation of effective mass.
